# A Tarantula Bite

**Published:** 1889-08

**Authors:** J. C. Baird

**Affiliations:** Pidcock’s Ranch


### A TARANTULA BITE.

BY J. C. BAIRD, M. D., PIDCOCK'S RANCH.

(For Daniel's Texas Medical Journal.)

J. W., age 38, stout and hearty. About 3 o'clock, a. m., May 9th, felt something crawling over his breast, "grabbed" and crushed it. Did not get up yet, the pain was such that he did not sleep any more. Got up early and went to work; thought that he had been bitten by a wasp, and that he would soon be all right. By noon the parts were swollen, and quite painful; at which time, the children told him that a large black spider was on the floor, near his bed, mashed up. Then he sent to me for "something to put on a spider bite." I sent him a solution of permang. pot., which I generally use for such cases, thinking nothing more it until my return Saturday, at noon, this being Thursday, when I was summoned to see Mr. W. in haste.

On my arrival, found patient in bed; said he was too sick to stand up, and that he had not slept or rested five minutes since he was bitten, the pain being very severe all the time, of a burning, lancinating character with extreme nausea. Had eaten nothing; bowels not acted since the bite; kidneys acted but very little; pulse fast and weak; cold clammy sweat; pupils dilated; expression that of extreme anguish and pain. The bite, about two inches above right nipple, and from the wound extending about 3 by 6 inches, was a very dark place, swollen and very painful; entire body swollen; right arm paralyzed; body of a dark mottled appearance.

The treatment: First I gave morph. ar. spts. ammonia and alcohol. Then cast. oil and turpentine for the bowels; in half hour began tr. iodine, ten drops with the stimulant, one hour apart, until kidneys act well. Then three hours apart, locally, laudanum in solution carb. amm. warm, this to be kept up until my return at 5 p. m. Found patient sitting up in bed, feeling some better, kidneys acted freely, but not the bowels. The dark place about the wound was now of a yellowish green appearance, entire chest very red and tender, looking very much like erysipelas, temperature 104, but pulse still weak, cold sweating had stopped, skin hot and dry. Gave antipyrin 10 grs. every two hours until fever subsides. Sulph. magn. for the bowels; continue tr. iod. with stimulants, three hours apart, stimulant oftener if needed. Locally, poultices wet with perm. pot. and laudanum. Next day found patient clear of fever, bowels and kidneys acting well, general swelling going down. Being satisfied that the dark place, three by six inches, was now entirely dead, or gangrenous, ordered charcoal with the poultices, and to relieve the pain about edges of dead flesh, used hydro, chl. cocoaine locally, and if need to secure rest, morph. at night. On Tuesday I removed the slough, and it left an ugly wound, fully half an inch deep; washed with carbolized water, then dusted with iodoform and boric acid finely powdered, edges of wound to be kept soft with carbolized oil, over which borated cotton to be kept all the time. This treatment has been kept up, lengthening time and lessening quantity as needed. Now, June 20, have a sore about 1 by 3 inches, healthy and free from pain more than would be from any ordinary sore; patient otherwise in usual good health. Brother M. D.'s, could the treatment have been improved, or would *nature* have been sufficient to overcome the poison, and prevent death without medical aid? Any criticism or suggestion will be appreciated. I have reported this to show the toxic effect short of death, as there is a doubt, seemingly, with writers, as to whether the tarantula is really poisonous or not.

NOTE.—*Dear Doctor:* Herewith I send you report of a case of tarantula bite. This line of treatment I generally use in snake bites, never having seen any one bitten by a tarantula before.

Last week I was called in some 20 minutes after the occurrence. A man at night, while wading the creek near here, stepped on a moccasin, which bit him on the instep under the water. I first injected solution of pot. permanganate into the wounds; then applied carbolic acid pure to the wounds and ordered cloths wet with solution of pot. perm. to be kept up and 10 drops tr. iodine in a little toddy every hour for four doses. In six hours all swelling was gone and nothing save a little soreness and stiffness in the ankles. Now the question is, did being bitten *under the water* so modify the poison that it was of little consequence? as I have never before had a bite get well so speedily.

